# Rewiring the evolution of the human hand: How the embodiment of a virtual bionic tool improves behavior

**DOI:** 10.1016/j.isci.2024.109937

**Published:** 2024-06-06

**Authors:** Matteo Marucci, Ottavia Maddaluno, Colleen Patricia Ryan, Cristina Perciballi, Simona Vasta, Simone Ciotti, Alessandro Moscatelli, Viviana Betti

**Affiliations:** 1 Department of Psychology, Sapienza University of Rome, Rome, Italy; 2 Laboratory of Neuroscience and Applied Technology, Santa Lucia Foundation IRCCS, Rome, Italy; 3 Department of Systems Medicine, University of Rome Tor Vergata, Rome, Italy; 4 Laboratory of Neuromotor Physiology, Santa Lucia Foundation IRCCS, Rome, Italy; 5 Information Engineering Department and the Research Center “E. Piaggio”, University of Pisa, Pisa, Italy

**Keywords:** Behavioral neuroscience, cognitive neuroscience, Bionics

## Abstract

Humans are the most versatile tool users among animals. Accordingly, our manual skills evolved alongside the shape of the hand. In the future, further evolution may take place: humans may merge with their tools, and technology may integrate into our biology in a way that blurs the line between the two. So, the question is whether humans can embody a bionic tool (i.e., experience it as part of their body) and thus if this would affect behavior. We investigated in virtual reality how the substitution of the hand with a virtual grafting of an end-effector, either non-naturalistic (a bionic tool) or naturalistic (a hand), impacts embodiment and behavior. Across four experiments, we show that the virtual grafting of a bionic tool elicits a sense of embodiment similar to or even stronger than its natural counterpart. In conclusion, the natural usage of bionic tools can rewire the evolution of human behavior.

## Highlights

• In the next future humans may merge with tools integrating technology into biology
• We created a novel type of tool virtually grafted to participants’ body
• We tested whether healthy participants can embody this bionic tool
• Participants embody a non-anthropomorphic tool better or equally than a virtual hand

## Graphical abstract

**Figure:**
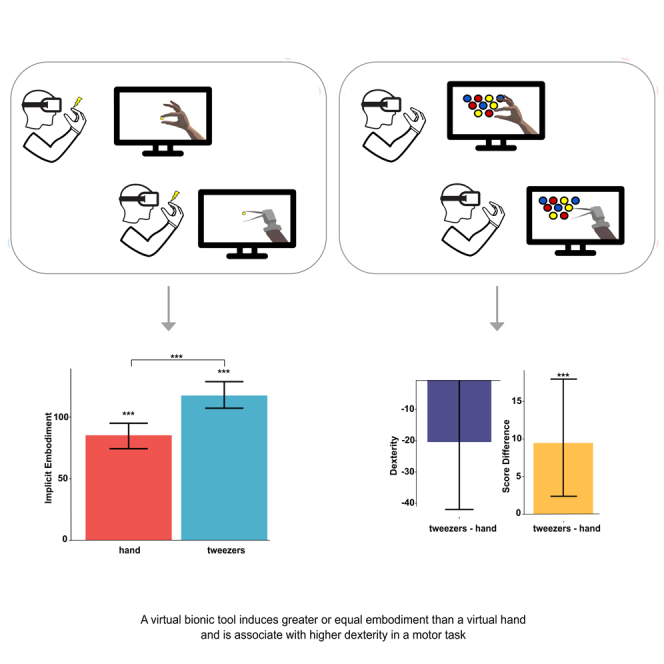

## Introduction

The unique capabilities of human hands have played a crucial role in our evolutionary success.^1^ Throughout phylogenesis, the shape of the human hand has specifically evolved to master prehensile and manipulation abilities, allowing us to interact with and shape the environment.^2^ Furthermore, the exceptional ability of humans to craft and utilize tools is enabled by the dexterous control of hands.^3^^,^^4^ Over time, our extensive and skillful use of tools shaped our behavior and influenced how our bodies perceive and interact with the world.^5^^,^^6^

In parallel with the unique expertise in tool use, the human brain developed sophisticated circuits to master the motor control of tools.^7^^,^^8^^,^^9^ Lesions to these circuits can lead to neurological deficits (e.g., apraxia) characterized by the inability to use objects and tools.^10^ Tool usage can modify these circuits, as demonstrated in animals. For example, monkeys trained to use a simple tool (a rake) to retrieve food, showed plastic changes in the visual receptive fields (RF) of bimodal neurons devoted to the representation of the space around the hand.^11^^,^^12^ Such expansion of the visual RF has been interpreted as a neural substrate of the assimilation of the tool into the body schema.^12^

Two decades of research have shown that tool use has also a profound impact on motor control^13^^,^^14^ and the representation of the body^15^^,^^16^^,^^17^ (i.e., the body schema, an internal model of the body which subserves action). For example, in humans, short periods of tool use induce an extension of the perceived arm length and modulate the movements of a free hand, influencing acceleration, deceleration, and velocity peaks.^13^^,^^14^ These results have been interpreted as the modulation of the body schema,^11^^,^^12^^,^^13^ that it can even occur after the mere imagination of tool usage.^18^ Notably, tool usage also elicits a sense of embodiment.^19^^,^^20^^,^^21^ The sense of embodiment consists of what it is like to have a body, and includes the sense of ownership among other experiences.^22^^,^^23^ In the framework of tool usage, embodiment refers to the subjective experience that an external object, such as a tool or prosthetic device, is perceived as an integral part of one’s own body.^19^^,^^20^^,^^21^

Prosthetic devices can be considered as a special class of tools for their effect on embodiment and body schema.^24^^,^^25^ Developments in technology allowed the grafting of robotic prostheses, in the replacement of lost limbs.^26^^,^^27^ An open scientific question is whether humans can embody non-anthropomorphic bionic tools, and treat them as part of their own body. Embodied bionic tools will allow acquired amputees to execute complex tasks with precision tools (e.g., goldsmiths, dentists) with the same ease and dexterity. This technological advancement will overcome the limitation of current prosthetic devices in daily living activities.^28^ Understanding if the embodiment of a non-anthropomorphic tool is possible and whether it allows a dexterous motor control is fundamental for the development of next generation robotic prostheses.

Embodiment plays a crucial role in the dexterous use of tools^12^^,^^29^ and it may improve usability and control of wearable technologies. This raises an intriguing question: can humans symbiotically merge with a bionic tool replacing their hand? This idea is grounded on the availability of innovative technological applications, ranging from augmentative robotics (e.g., the sixth finger^30^ or third thumb^31^) to brain-, muscle- and body-machine interface systems.^32^ These interfaces enable the control of non-anthropomorphic bionic limbs intuitively and seamlessly. To address this question, in healthy participants, we manipulated the appearance of their hand in virtual reality (VR). We replaced participants’ right hand with a virtual effector, either naturalistic (i.e., a virtual hand) or non-naturalistic (i.e., a virtual bionic tool), and investigated the impact on embodiment and motor behavior. Our hypothesis is that humans can experience a bionic tool as part of their own body and use it effectively. To test this idea, the embodiment and the performance in a motor task using the bionic tool were compared to those obtained with a virtual hand (baseline condition). We performed four experiments using implicit (experiments 1–3) and explicit (experiment 4) measures of embodiment. Implicit measures are based on automatic responses and are less susceptible to cognitive and response biases. An example is the Crossmodal Congruency Task (CCT).^33^ CCT is a well-known visuo-tactile paradigm that, by assessing the interplay between a tactile stimulus and a visual distractor, provides an implicit measure of tool-related change in the sense of body ownership,^34^^,^^35^^,^^36^ the peripersonal space (the space immediately surrounding the body) and embodiment.^25^^,^^37^ In CCT, participants are presented with short pulses of vibrations (tactile stimuli) presented to different fingers of the same hand and are requested to report on which finger the stimulus was delivered. A visual distractor (visual stimuli) consisting of a brief flickering stimulus is displayed either on the same or a different finger as the tactile stimulation. Congruent stimuli are typically associated with shorter response times and fewer errors, as compared to incongruent stimuli. This effect is known as the crossmodal congruency effect (CCE). Previous studies used CCE as an implicit measure of embodiment in the rubber hand illusion^34^^,^^35^ and prosthetics.^25^ Likewise, CCE can be elicited by visual distractors at the tip of a hand-held tool.^38^ Additionally, explicit measurements such as surveys and self-report questionnaires are used to assess the embodiment of a rubber hand or a tool since they are based on conscious thoughts, beliefs, and behaviors.^22^^,^^23^^,^^39^^,^^40^

We used the CCT as an implicit measure of ownership either alone (experiment 2–3) or interspersed by blocks of motor task (MT) (experiment 1,4). Existing literature demonstrates that the active movement of a virtual hand increases the sense of body ownership in VR.^41^^,^^42^^,^^43^ Therefore, the aim of the MT was two-fold: to assess the motor performance (measured as reaction time and number of errors) and to induce a higher degree of embodiment of the virtual hand and the bionic tool.

## Results and discussion

**Figure 1:**
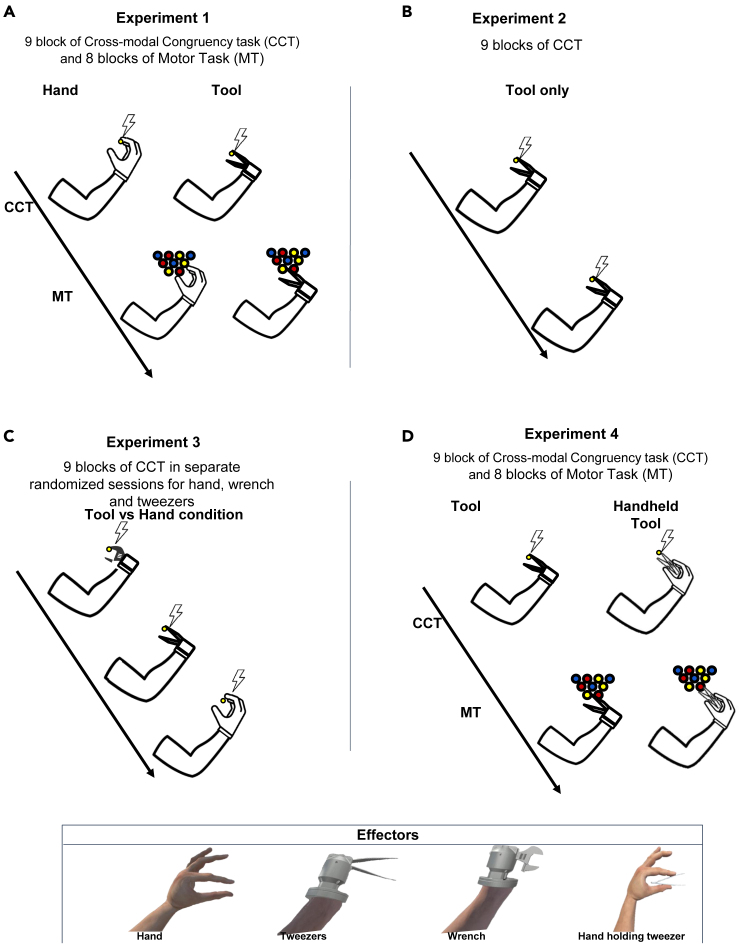
Depiction of the experimental designs (A) In experiment 1 participants underwent 8 blocks of MT and 9 blocks of CCT. They performed the tasks either with the virtual hand or the virtual bionic tool (i.e., tweezers). The order of the sessions was counterbalanced between participants. (B) In experiment 2, participants performed only the CCT with the tweezers (9 task blocks). (C) During experiment 3, participants performed only the CCT with 3 different virtual effectors: i.e., tweezers, wrench, or hand. The order of the sessions was counterbalanced across participants. Panel at the bottom, images of the virtual hand, tweezers and wrench. (D) In experiment 4, participants performed the CCT and the MT (as in experiment 1) using either a bionic tool or a virtual hand holding a pair of tweezers.

Here, in four different experiments (total number of participants N = 87, each participant took part to one of the four experiments; see STAR methods) we tested the hypothesis that a virtual bionic tool can be experienced as a part of our own body, as assessed with well-established implicit and explicit measures of embodiment, and we compared this level of embodiment with the one elicited by a virtual hand. In accordance with this hypothesis, using VR, we found that a virtual bionic tool (labeled as tweezers or wrench in Figure 1) elicits in the participants a sense of body ownership. As we demonstrate, the higher level of embodiment allows participants to perform tasks with greater precision and ease when using the bionic tool than with a virtual hand. In experiment 1, we investigated the embodiment of two different effectors: i.e., a virtual hand and a virtual bionic tool. We used an implicit paradigm, i.e., a modified version of the Crossmodal Congruency Task (CCT^33^^,^^44^; see STAR methods), to assess, in virtual reality (VR) the embodiment of the bionic tool and the virtual hand. During the CCT, a light flashed in correspondence with the index or the thumb of the virtual hand, or to the upper or lower branch of the tweezers, respectively. Simultaneously, a small vibration was delivered to the same (congruent trials) or the other digit (incongruent trials) of the participants’ hand through a haptic device. Participants were required to report on which digit the vibration was felt, ignoring the visual distractor (i.e., the light stimulus).

**Figure 2:**
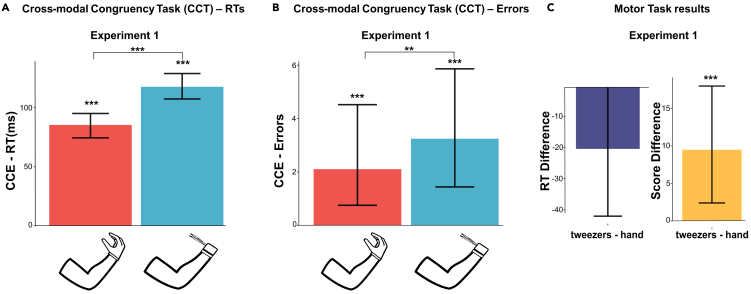
Experiment 1: Embodiment of the two effectors (A) Mean Crossmodal Congruency Effects (CCEs; performance on incongruent trials minus performance on congruent trials) for Response Time (RT). RT were shorter in congruent than in incongruent trials (CCE). Moreover, the CCE was larger with the tweezers as compared to the hand (circa 85 ms slower). (B) Mean CCE for the number of errors during the two conditions (i.e., bionic tool and naturalistic hand). The number of errors is higher in incongruent trials for the tweezers condition. (C) Results for MT: differences in response time and score for tool condition minus hand condition. RTs are faster when participants use a pair of tweezers. On average, participants reach a higher score during the bionic tool condition. Data are represented as mean ± SEM. (∗p < 0.05, ∗∗p < 0.01, ∗∗∗p < 0.001, linear mixed model and general linear mixed model).

In the classical CCT, the performance is worse in incongruent trials than in congruent trials if the participant embodies the effector.^35^ This difference in performance between incongruent and congruent trials, known as the crossmodal congruency effect (CCE), estimates the influence of the visual distractor on discriminating the tactile target location, and it is a well-established implicit measurement of the sense of body ownership of the virtual or real prosthesis.^34^^,^^44^ Therefore, by evaluating the CCE, it was possible to compare the embodiment between the virtual hand and the virtual bionic tool. To strengthen the functional similarities between the two virtual end-effectors, we also implemented a Motor Task (MT) in which participants were required to pop a sphere of a target color that appeared among a group of distractors. Short blocks of CCT and MT were interspersed within the same experimental session (refer to STAR methods; Figure 1A). We used a linear mixed model to analyze reaction times (RT) during the CCT. The RT-CCE — equal to the difference in RT between incongruent and congruent trials — was larger with the virtual tool (117.5 ms slower in incongruent trials, 95% CI from 107.9 to 128.2 ms) as compared to the hand (85.3 ms slower in incongruent trials, 95% CI from 74.9 to 95.4 ms) (Figure 2A). Crucially, this difference in RT-CCE between the two end-effectors was statistically significant (Estimate = 32.24, Std. Error = 7.4, p < 0.001). Although the RT-CCE was higher with the bionic tool, also when the virtual effector was the hand the RTs were higher in the incongruent trials with respect to congruent trials (Estimate = 85.3 ms, Std. Error = 5.2, p < 0.001). Results also show a significant main effect of the tool vs. hand on RT (Estimate = −23.15, Std. Error = 5.4, p < 0.001). This means that in the two types of trials participants were faster with the virtual bionic tool compared to the virtual hand. Using General Linear Mixed Models, we tested whether the error count was different between trial types depending on the effector, (Error-CCE). Remarkably, the interaction between effector and trial type was statistically significant (Estimate = 0.84, Std. Error = 0.36, p = 0.019) meaning that the Error-CCEs were higher while using the bionic tool (the model predicted 3.2 more error in incongruent trials, 95% CI from 1.7 to 6.1) instead of the hand (2.1 more errors in incongruent trials, 95% CI from 0.8 to 4.7) (Figure 2B). The main effect of effector (hand vs. bionic tool) was not statistically significant (Estimate = - 0.58, Std. Error = 0.36, p = 0.11). See supplemental information (Figures S1 and S2), where we provided, for the CCT task, mean and confidence intervals of the RT values and Errors for each condition (bionic congruent, bionic incongruent, virtual hand congruent and virtual hand incongruent). Taken together, the positive values of RT-CCE and the Error-CCE suggest the embodiment in both anthropomorphic (i.e., resembling a hand) and non-anthropomorphic (i.e., a bionic tool) prosthesis (higher RT and more errors in the incongruent trials). Strikingly, the embodiment of the bionic tool was stronger compared to the one induced by a virtual hand, as shown by the difference in RT-CCE and Error-CCE between conditions.

Next, we analyzed the errors and RT in the motor task. Participants committed less errors with the tool as compared to the hand (Estimate = 0.05, Std. Error = 0.02, p = 0.023), suggesting a higher dexterity using the former (Figure 2C, right). Results also show a non-significant trend on RT. Participants were slightly faster to pop the spheres with the tool (Estimate = −20.44, Std. Error = 11.22, p = 0.07) (Figure 2C, left).

### Factors underlying embodiment of the virtual bionic tool

**Figure 3:**
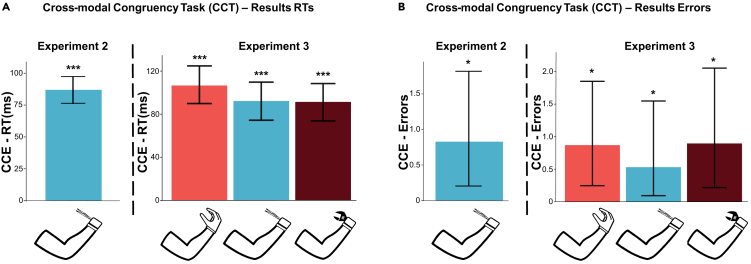
Experiment 2 and 3: Factors underlying embodiment (A) Mean CCE for response times. Experiment 2 confirmed that participants are slower in incongruent trials when using a pair of tweezers. In experiment 3 we investigated if the morpho-functional properties of the tool modulate the embodiment. On average RTs were slower in incongruent trials during the virtual hand condition. (B) Mean CCE for errors number. During experiment 2 results show a higher error rate for incongruent trials. Taken together, this evidence suggests that participants embodied the bionic tool. In experiment 3, participants commit more errors in incongruent trials while performing the task with the virtual hand, thus it seems that the motor task is necessary to evoke higher embodiment in the bionic tool as compared to a virtual hand. Data are represented as mean ± SEM. (∗p < 0.05, ∗∗p < 0.01, ∗∗∗p < 0.001, linear mixed model and general linear mixed model).

In the first experiment, the motor task may have contributed to the sense of ownership toward the virtual effectors. Since we do not have specific reasons to assume that this effect was different across the virtual bionic tool and the hand, we performed a second experiment (Figure 1B) by asking participants to perform only the CCT with the bionic tool (i.e., the condition that elicits more embodiment). Here, the aim is to test whether the visual appearance is sufficient to elicit a sense of embodiment. As in the first experiment, analysis of RTs showed that participants were slower (Estimate = 116.48, Std. Error = 8.84, p < 0.001) during incongruent trials (Figure 3A, left). The barplot shows the RT-CCE which is significantly different from zero. The estimate between the two experiments was similar (117.5 ms slower in incongruent trials, 95% CI from 98.1 to 133.0), confirming the embodiment of the tool. Consistently, the number of errors was higher in incongruent trials (0.82 more errors; 95% CI from 0.20 to 1.80) as also confirmed by the effect of congruency in the GLMM (Estimate = 1.91, Std. Error = 0.82, p = 0.0197) (Figure 3B, left). Both RT-CCE and Error-CCE suggest that the embodiment of the virtual bionic tool is present even without the motor task.

Since the morphofunctional characteristics of the tool may determine our results, in the third experiment, we introduced a novel bionic tool (similar to a wrench) to test the generalizability of the embodiment to another virtual bionic tool, differentiating from the tweezers for the visual and morphofunctional properties. Here, participants performed only the CCT (without the motor task) with three possible virtual effectors: the hand, a pair of tweezers, and a wrench (Figure 1C). Results show that RTs were slower during incongruent trials while performing the task with the virtual hand (Estimate = 106.63, Std. Error = 8.39, p < 0.001) (Figure 3A, right). Notably, in the third experiment the two-level interaction between trial type and the two effector conditions was not statistically significant neither for the tweezers (Estimate = −14.38, Std. Error = 12.05, p = 0.23) nor for the wrench (Estimate = -15.22, Std. Error = 12.03, p = 0.21). We also found a main effect of tweezers (on average RT 18 ms slower compared to the hand condition) (Estimate = 18.92, Std. error = 8.11, p = 0.019). The main effect of the wrench was not significant (Estimate = 2.80, Std. Error = 8.52, p = 0.74). Results on the error number mirrored the results on RTs: We observed more errors during incongruent trials in the hand condition (Estimate = 2.06, Std. Error = 0.82, p = 0.01), and the interaction between trial type and tool was not statistically significant (meaning that the effect of congruency was similar across the three end effectors). The Error-CCE was equal to 0.87 in the hand condition, 0.53 for the tweezers, and 0.89 for the wrench (Figure 3B, right). Overall, the results of the third experiment suggest that the consistent visuospatial localization between the hand and the virtual tool is a sufficient condition to elicit a basic level of embodiment of the effector. However, familiarizing with the bionic tool during the motor task is necessary to evoke a higher embodiment of it as compared to a virtual hand.

Overall, this first set of results highlights that humans can embody a tool, similarly or even more than a virtual hand.

Finally, in experiment 4 we investigated whether the presence/absence of the hand interacting with the tool modulates the embodiment (assessed through implicit and explicit measures). Therefore, in this experiment participants performed both the CCT and the MT while using either a virtual hand holding a pair of tweezers or a virtual bionic tool (as in experiment 1) (Figure 1D). A subset of this sample (N = 17) underwent a questionnaire^39^ to assess the embodiment of the two conditions.

**Figure 4:**
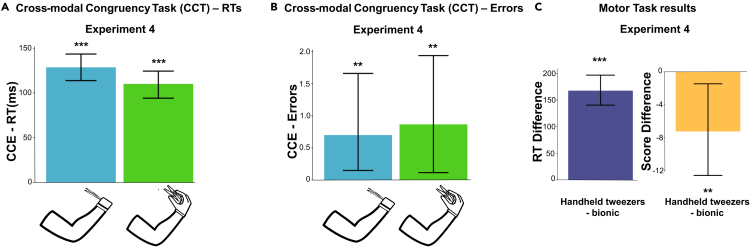
Experiment 4: different level of body manipulation through VR (A) Mean CCE for response time. RT were shorter in congruent than in incongruent trials (CCE). The CCE was slightly bigger with the bionic tool as compared to the hand holding the tweezers. (B) Mean CCE for the number of errors during the two conditions. The number of errors is higher in incongruent trials for the tweezers condition, but the effect is not significant. (C) Results for MT: differences in response time and score for handheld tweezers minus bionic tool condition. RTs are slower when participants use a virtual hand holding the tweezers. On average, participants reach a slower score during the handheld tweezers condition. Data are represented as mean ± SEM. (∗p < 0.05, ∗∗p < 0.01, ∗∗∗p < 0.001, linear mixed model and general linear mixed model).

Results in CCT show that RTs were higher during incongruent trials (Estimate = 128.61, Std. Error = 7.12, p < 0.0001). Results show a non-significant trend for the interaction between end-effector and trial type. That is, the RT-CCE was smaller when using the virtual hand holding the tweezers compared to the bionic tool (Estimate = −18.55, Std. Error = 10.11, p = 0.067) (Figure 4A). We also found a main effect of the hand with tweezers such that participants were 25 ms slower when performing the task with this effector, independently of the trial type (Estimate = 25.08, Std. Error = 6.73, p < 0.001).

The virtual end-effector seems not to influence the error number in the CCT (Figure 4B). Specifically, with the bionic tool, participants committed more errors in incongruent trials than congruent ones (Estimate = 2.41, Std. Error = 0.76, p < 0.01). The interaction between effector and trial type was not significant (Estimate = −1.03, Std. Error = 0.81, p = 0.21), meaning that the CCE-Errors were similar between the two conditions. We observe a non-significant trend such that, when using the virtual hand holding the tweezers participants committed more errors (Estimate = 1.43, Std. Error = 0.79, p = 0.069). As for the first study, for the CCT task, we provided mean and confidence intervals of the RT values and Errors for each condition (see supplemental information). Results on the MT are in accordance with the results of experiment 1: participants were faster popping the spheres (Estimate = 167.52, Std. Error = 14.15, p < 0.0001) and committed fewer errors (Estimate = −0.08, Std. Error = 0.03, p < 0.01) with the virtual bionic tool as compared to the hand with tweezers (Figure 4C). For a complete overview of the results of the four experiments, see Table 1.

**Table 1:** Results of the 4 experiments

	Independent	Predictor	Estimate	SE	p value
Experiment 1	RTs CCT	Intercept	677.78	33.83	<0.001∗∗∗
		Effector	−23.15	5.38	<0.001∗∗∗
		Trial Type	85.28	5.23	<0.001∗∗∗
		Effector ∗ Trial Type	32.24	7.37	<0.001∗∗∗
	Errors CCT	Intercept	−0.36	0.34	0.28
		Effector	−0.58	0.36	0.11
		Trial Type	1.39	0.34	<0.001∗∗∗
		Effector ∗ Trial Type	0.84	0.36	0.02∗
	RTs MT	Intercept	1906.67	57.64	<0.001∗∗∗
		Effector	−20.44	11.22	0.07
	Score MT	Intercept	5.17	0.02	<0.001∗∗∗
		Effector	0.05	0.02	0.03∗
Experiment 2	RTs CCT	Intercept	844.57	51.41	<0.001∗∗∗
		Trial Type	116.48	8.84	<0.001∗∗∗
	Errors CCT	Intercept	−1.94	0.84	0.02∗
		Trial Type	1.91	0.82	0.02∗
Experiment 3	RTs CCT	Intercept	801.83	50.05	<0.001∗∗∗
		Hand vs. Tweezers	18.93	8.11	0.02∗
		Hand vs. Wrench	2.80	8.52	0.74
		Trial Type	106.63	8.39	<0.001∗∗∗
		HandTweezers ∗ Trial Type	−14.38	12.05	0.23
		HandWrench ∗ Trial Type	−15.22	12.03	0.21
	Errors CCT	Intercept	−2.06	0.81	0.01∗
		Hand vs. Tweezers	−0.82	0.76	0.28
		Hand vs. Wrench	−0.48	0.69	0.48
		Trial Type	2.06	0.82	0.01∗
		HandTweezers ∗ Trial Type	0.29	0.70	0.68
Experiment 4	RTs CCT	Intercept	651.41	32.10	<0.001∗∗∗
		Effector	25.08	6.73	<0.001∗∗∗
		Trial Type	128.61	7.12	<0.001∗∗∗
		Effector ∗ Trial Type	−18.55	10.11	0.067
	Errors CCT	Intercept	−2.68	0.71	<0.001∗∗∗
		Effector	1.43	0.79	0.069
		Trial Type	2.41	0.76	<0.01∗∗
		Effector ∗ Trial Type	−1.03	0.81	0.21
	RTs MT	Intercept	1703	56.67	<0.001∗∗∗
		Effector	167.52	14.15	<0.001∗∗∗
	Score MT	Intercept	4.54	0.02	<0.001∗∗∗
		Effector	−0.08	0.03	<0.01∗∗

SE, standard error.

∗p < 0.05 ∗∗p < 0.01 ∗∗∗p < 0.001.

We used a Linear Mixed Model on RTs for the Crossmodal Congruency Task (CCT) and Motor Task (MT) and a General Linear Mixed Model to analyze errors and scores on the CCT and MT.

In summary, these final results suggest that participants were more dexterous and accurate in the motor task with the bionic tool as compared to the other end-effector. We also observed a small increase in the sense of embodiment (non-significant trend) of the tool compared to the hand holding the tweezers.

### Subjective questionnaires

**Figure 5:**
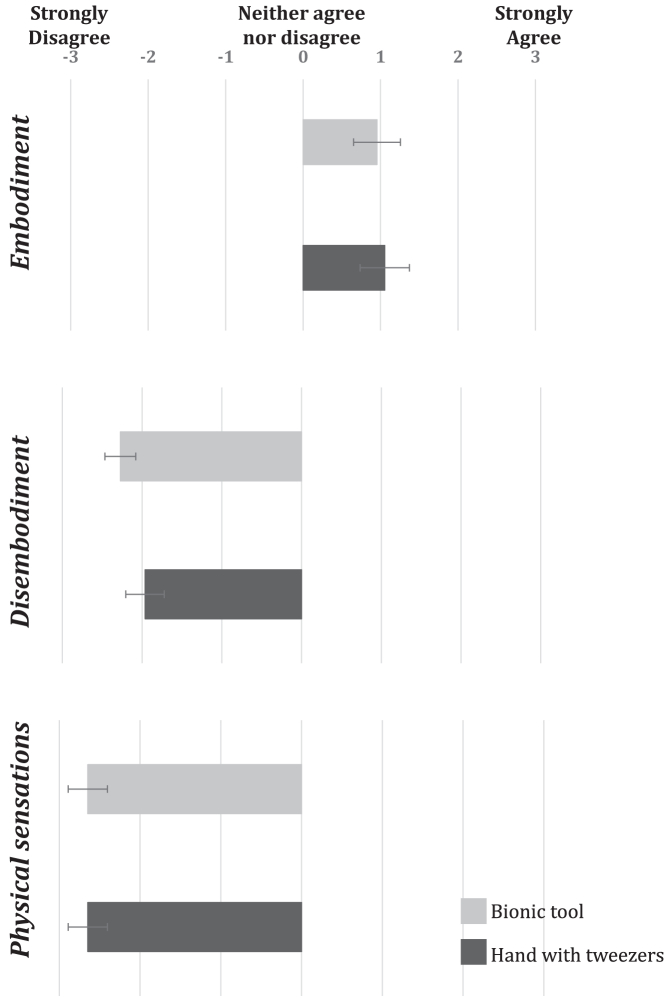
Explicit measure of embodiment Mean scores on the three questionnaire scales for the two conditions: i.e., bionic tool and hand holding the tweezers. Data are represented as mean ± SEM.

Regarding the questionnaire measures, we used a modified version of the questionnaire by^39^ that assesses embodiment on three different scales: i.e., Embodiment, Disembodiment (that is relative to the control and ownership of one’s own hand) and Physical sensations (that is pleasant/unpleasant sensations felt). Since the data were not normally distributed (Shapiro-Wilk p < 0.05), we analyzed the differences between the two effectors on the scales rating using a Wilcoxon test. Results on the Embodiment scale highlight how both effectors (the hand holding the tweezers and the virtual bionic tool) seem to elicit a certain level of embodiment (mean scores: hand with tweezers = 1.06, bionic tool = 0.95). However, there was no significant difference between the effectors (W = 89.5, p = 0.27). Ratings on the Disembodiment scale were no different between the two conditions (mean scores: hand with tweezers = −1.96, bionic tool = −2.27; W = 76, p = 0.15), meaning that participants did not experience any decrease in the sense of ownership of their own limb with either end-effector. Finally, there was no significant difference also on the Physical sensations scale (mean scores: hand with tweezers = −2.65, bionic tool = −2.65) (Figure 5 and supplementary information).

### General discussion

It is possible to broadly distinguish three types of interfaces that allow object interaction: 1) tools that people can grasp and manipulate with their intact hands, 2) anthropomorphic prostheses replacing the lost limb in amputees, and 3) augmentative robotic interfaces that extend or replace upper-limb abilities. In this study, we introduce a new virtual interface: a virtual bionic tool grafted to the participant’s virtual arm. This embodied tool is not anthropomorphic as a prosthesis, however it is integrated in the user’s body and can be virtually grafted in able-bodied participants. In this way, participants can translate biological movements of their fingers directly into the movement of an otherwise external tool. An extensive literature, testing the effects of tool usage, highlights that tools extend the representation of our body (e.g.,^45^). Here, we expand this field of research, virtually grafting a tool, aligned and shaped to the participants’ upper limb, to perform a motor task.

In the first experiment of the study, participants showed higher embodiment (Figures 2A, 2B, 3A and 3B), faster reaction time and less error when interacting with objects through a virtual bionic tool as compared to an anthropomorphic effector (Figures 2C–4C). Consistently with Zopf and colleagues^35^ in the real environment, in VR we observed a larger difference between congruent and incongruent trials during a crossmodal task, an implicit measure of embodiment. This result is consistent across the four experiments (Figures 2A, 2B, 3 and 4). The reasons underlying the successful embodiment and usage of the bionic tool can be associated with the complexity of the human hand, a sensorimotor system with more than 20 degrees of freedom.^46^^,^^47^^,^^48^^,^^49^ Based on these findings, these results may suggest that the embodiment of a prosthesis benefits from simplifying the control of such a complex distal effector.^49^ Since the neural circuits controlling the hand are naturally wired in a synergistic fashion, it will be possible to design a virtual bionic tool exploiting low dimensionality toward a simplified control of the prosthesis. Although the synergy model has been already used for the design of prostheses and artificial hands,^50^ even the latest technologies are still aesthetically similar to their natural counterparts.^51^ By extending this view, in this study, we demonstrate that the embodiment can be even higher for effectors that are visually far different from the hands, yet they mirror its function (hand aperture-closure). It should be noted that the tracking system employed also allowed wrist flexures to be reflected on the 3days models of the hand and tool in the same way. This means that there is no facilitation in embodiment levels attributable to differences in the virtual reproduction of kinematics.

Traditionally, embodiment has been studied using the well-known paradigm of the Rubber Hand Illusion (RHI), through which participants feel the illusion that a rubber hand belongs to their body. This paradigm is effective in eliciting a sense of ownership.^22^^,^^52^ In addition to the rubber hand, the use of tools also shapes the peripersonal space,^37^^,^^53^^,^^54^^,^^55^^,^^56^^,^^57^ the body schema^13^^,^^15^^,^^16^^,^^24^^,^^56^ and alters hand kinematics.^13^^,^^14^^,^^18^^,^^58^^,^^59^ Inspired by the experiments on macaque monkeys^11^^,^^12^ studies on humans evaluated the modulation of multisensory integration and body representation during tool usage.^15^^,^^16^^,^^17^^,^^60^ For instance, evidence showed that short training on tool use changes the body metrics. Such alterations in the perceived size or length of the body, represent evidence of the so-called representational plasticity induced by tool use.^17^^,^^61^ This kind of plasticity can occur also at the neuronal level as suggested by the modulation of the space around the hand after tool use.^11^^,^^62^ Overall, these results suggest that tools can be incorporated into our body schema. Recent studies aim to explicitly assess the embodiment of a tool with both explicit and implicit measures.^19^^,^^20^^,^^21^ For example, Cardinali and colleagues used a modified version of the rubber hand illusion in which a mechanical grabber is stroked instead of a rubber hand. Their results show modifications of both implicit (e.g., perceived localization of their own hand and physiological response when the tool was threatened) and explicit measures of embodiment (feeling of the grabber as being part of participants’ body).

Crucially, all these studies employ hand-held tools. By contrast, here we measure the embodiment of a virtual tool directly grafted to the participant’s arm. This brand new paradigm paves the way to novel interesting considerations. Here, we found that the bionic tool elicits equal or even more embodiment of a virtual hand (e.g., exp 1). This result can be explained by the fact that the movement of the real physical hand is directly translated into the morphofunctional features of the tool (e.g., tweezers). In other words, the sensorimotor (the input) and the morphofunctional features (the output) are aligned, spatially overlapping, and directly translated into the movement of the tool. Our interpretation is that the aesthetic characteristics of the virtual hand can reduce the affinity with the effector, consistently with the “uncanny valley effect.”^63^^,^^64^ According to the uncanny valley hypothesis, the affinity with human-like robots, hand-like prostheses, or avatars, is reduced within a specific range (close but not enough) of human likeness.^63^ Thus, our interpretation is that our virtual hand, even if realistic, does not sufficiently resemble the participants’ real hand. This is consistent with a recent study in VR showing that the subjective ownership of a 3days scanned hand was stronger than that of a virtual fake hand.^65^

Another important aspect is the role of the motor task. Even if our study is not primarily designed to test the causal relationship between the motor task and the sense of ownership, it is reasonable to assume that the embodiment improved the dexterous control of the virtual effector. On the other hand, the motor task can facilitate the sense of body ownership. Notably, movements of the virtual effector mirrored the ones performed by the participant’s real hand. This matching has already been demonstrated to be a crucial factor for the sense of agency (the feeling of control and influence over the external tool).^19^^,^^66^ The importance of the role of agency in the construct of embodiment has been supported by recent studies. Within the context of tool usage, previous studies highlight how the active use of a tool leads to high levels of embodiment^18^ and that the passive holding of it fails to alter body schema and induce the perception of the tool as part of one’s own body.^67^^,^^68^ However, it is worth noting that the results of our second experiment demonstrated the embodiment of the virtual bionic tool, to some extent, also in the absence of an active motor task. That is, the difference between congruent and incongruent trials was still present when using the virtual bionic tool, highlighted by slower reaction times during incongruent trials. Thus, the mere observation of the bionic tool is sufficient to induce a sense of ownership. This is consistent with recent studies (e.g.,^69^).

The development of new wearable technologies is crucial for the advancement in the prosthetics field. Usually, prostheses focus on mirroring the visual appearance of the lost limb, but still even the more sophisticated devices are not well embodied. One of the main reasons could be that they are not functionally useful for patients. Indeed, our results confirm that. Results of the first experiment showed that a tool, visually distinct from a human hand but in some ways functionally similar, is incorporated better than a virtual hand. One possible interpretation is that the facilitation effect is due to the morpho-functional characteristics of the virtual bionic tool. This is consistent with^16^ demonstrating how the similarity between the tool and the body part (e.g., a hand or an arm) is crucial to alter the body representation of the same part. We speculate that the visuo-spatial congruency and the similar functionality (i.e., grasping) between the position of the fingers and the moving parts of the tool (i.e., the tweezers’ tips) could have a role to this effect. In experiment 3, we tested this idea, by also including a wrench, whose moving parts are much shorter and less finger-like than tweezers. We confirm previous results on CCT on the different effectors. However, in the absence of a motor task, we did not find any advantage of the virtual tools compared to the hand. Crucially, in experiment 4, we confirmed that participants were more dexterous (in terms of accuracy and faster reaction times) when performing a motor task using the bionic tool even if compared to the virtual hand holding tweezers. This is important, as we extended the results of the first experiment and demonstrated that the virtual bionic tool is more effective than the hand holding the tool, even if this is the more natural condition.

Overall, these findings pave the way for a future research area in prosthetic development. In the near future, non-anthropomorphic robotic end-effectors may increasingly replace current prosthetic hands. This idea is not so far from reality. Examples are already present in the field of robotic-assisted surgery (e.g., Da Vinci System, Intuitive Surgical Inc., USA). For this reason, knowledge about factors underlying the successful use and embodiment of such technology is fundamental. Current knowledge indicates only 44% of amputees wear their prosthesis regularly.^70^^,^^71^^,^^72^ There are several reasons underlying prosthesis refusal.^73^^,^^74^ One of the main reasons is that amputees do not feel the prosthesis as one part of their own body (i.e., a lack of embodiment).^75^

The lack of the sense of ownership toward the prosthesis cohabits with phantom pain sensations of the missing limb due to the preserved structural and functional organization of the somatosensory brain regions of the missing limb.^76^^,^^77^ Research in the field is currently focusing on the development of rehabilitation protocols to reduce phantom pain and increase embodiment.^69^^,^^78^^,^^79^ Another line of evidence suggests the beneficial effects of multisensory stimulation. For example in a modified version of the rubber hand illusion, visual feedback combined with electro-cutaneous stimulation has proven to be effective in inducing the embodiment of a virtual leg and reducing phantom limb sensations in three lower limb amputees.^69^ These studies highlight the primary role of crossmodal stimulation in helping prosthesis embodiment. These advancements, together with the idea of the non-anthropomorphic effectors, can stimulate the employment of new prosthetic devices in real life scenarios, providing novel opportunities to investigate plastic changes in the structural and functional brain reorganization, likely a neural substrate underlying the increment of the embodiment and reduction of the phantom sensations (including pain). Moreover, further research is needed to investigate this issue.

Our findings suggest novel and intriguing lines of research and applications. On one hand, the possibility to embody tools has interesting clinical implications especially if we consider amputees struggling to embody anthropomorphic prosthesis.^74^ Moreover, the possibility of replacing the hand with non-anthropomorphic effectors may stimulate future industrial applications based on augmentative reality. Literature on robotics and supernumerary limbs has already demonstrated how these devices could improve performance.^80^ While the embodiment of a tool depends on a delicate interplay among morpho-functional characteristics, recent usage, expertise, multisensory stimulation and agency, it is important to underline how a high level of embodiment or performance does not automatically imply high levels of compliance. In fact, the use of a prosthetic device also relies on other factors, such as body image satisfaction, psychosocial adjustments and lack of activity restriction.^81^

On the other hand, we demonstrated that virtual reality could be used as a benchmark to explore the embodiment that each prototype offers to a specific person before building it. More interestingly, according to the view that the hand is a cognitive organ and its embodiment underlies the development of human behavior, skills, and cognitive functions,^82^^,^^83^ it is reasonable to expect the emergence of plastic phenomenon underlying the successful embodiment and usage of the virtual tool effector. This is in accordance with recent studies showing the emergence of new corticospinal motor synergies using augmentative robotic tools.^84^ Although this study is not properly designed to explore long-term effects, this issue remains of high topicality for future studies.

A final note should be taken regarding our findings on the explicit measure of embodiment (Figure 5). We found a dissociation between implicit (CCE) and explicit (questionnaire) measures of embodiment. However, this is not surprising. Even if questionnaires have been widely used to measure embodiment in the past, recently some authors pointed out how these measures can be biased by participants’ level of susceptibility.^85^ For example, Marotta and colleagues showed how scores on susceptibility scales highly correlate with the rating on a questionnaire measuring ownership of the rubber hand paradigm. Conversely, susceptibility level does not correlate with an implicit measure (i.e., proprioceptive drift). For this reason, implicit measures, such as the CCT, are considered more robust and are nowadays commonly used to investigate the sense of ownership.^33^^,^^34^^,^^35^ Moreover, some authors have recently started to develop new implicit measures to assess embodiment.^86^

Collectively, our findings demonstrate the adept human ability to experience a grafted tool, observed from a first-person perspective, as an integral part of their own body. Remarkably, the degree of the embodiment elicited by the bionic tool is equal or even higher than that elicited by a virtual hand.

### Limitations of the study

Despite our 3D hand model being photorealistic (see Figure 1), in the future it may be possible to investigate CCE with a model of the hand reproducing the unique features of each participant’s hand. Recently, it has been found that subjective ownership of participants’ 3days scanned hand observed in VR was stronger than that of a general 3days hand, both in congruent locations after synchronous stimulation.^65^

Importantly we preserved the hand kinematics, while visually replacing the hand with a virtual counterpart explored in first person perspective (similar to^87^). Therefore, even if participants were instructed to observe the tweezers or the virtual hand without moving, the infrared hand tracking camera would keep track of the occasional movements of the wrist and the fingers and update the virtual counterparts accordingly. This could have contributed to creating a sense of embodiment through a perceived agency toward the movements of the virtual effectors.

A third important limitation is that we cannot generalize our results to real-life situations. Further studies involving physical non-anthropomorphic bionic tools are necessary to better understand their embodiment in real-life settings.

## STAR★Methods

### Key resources table

**Table:** 

REAGENT or RESOURCE	SOURCE	IDENTIFIER
**Deposited data**		

Behavioral Data	This paper	https://osf.io/avxwp/?view_only=07f4583b02004ab7a16942ec3642e66d

**Software and algorithms**		

R	R Core Team	https://www.r-project.org/
Unity 3D	Unity Technologies	https://unity.com/

### Resource availability

#### Lead contact

Further information and requests should be directed to and will be fulfilled by the lead contact, Viviana Betti (viviana.betti@uniroma1.it).

#### Materials availability

This study did not generate new unique reagents.

#### Data and code availability

• The data have been deposited at Open Science Framework (OSF) and are publicly available. The link is listed in the key resources table.
• All original codes to analyze the data have been deposited at Open Science Framework (OSF) and are publicly available. The link is listed in the key resources table.Any additional information required to reanalyze the data reported in this paper is available from the lead contact upon reasonable request.

### Experimental model and study participant details

#### Supplemental experimental procedures

##### Subjects

A total of 87 Italian healthy human participants with normal or corrected-to-normal visual acuity were recruited in this study and distributed as follows: Experiment 1, 22 participants (14 male, mean age ±sd: 25 ± 3.4 years old); Experiment 2, 20 participants (8 male, mean age ±sd: 25.2 ± 4.2 years old); Experiment 3, 20 participants (6 male, mean age ±sd: 24.3 ± 4.06 years old); Experiment 4, 25 participants (8 male; mean age ±sd = 26,8 ± 4 years old). The sample size of each experiment is in accordance with previous studies using the cross-modal congruency task.^34^ Furthermore, we performed the power calculation as suggested in (Cohen 1988).^88^ Assuming a difference in RT between congruent and incongruent trials of 130 ms and an SD of 100 ms (as in^34^) the sample size for a power of 80% is equal to 7 participants and is equal to 15 participants if the effect size was reduced to 80 ms. Therefore, to further increase the statistical power, we increased the sample size to a numerosity of 20 participants or more, for each experiment. An important criterion of exclusion was hand dominance, that is, only right-handed people participated in the study. The experimental protocol was approved by the ethics committee of Sapienza University of Rome and was carried out under the ethical standards of the Declaration of Helsinki of 1964. All participants gave their written and signed informed consent before the experiment.

### Method details

#### Experimental setup

A virtual scenario consisting of an office was implemented in Unity3d 2019.4.16 using models downloaded from the asset store. Four avatars (Figure 1) have been obtained from a 3D model downloaded from mixamo (www.mixamo.com), the first model was completely the same as a human body, the second had instead of the right hand a bionic tool with tweezers in place of index finger and thumb and was rigged and modified using Blender (www.blender.org). For the third avatar, we used a modified version of the second model with a wrench tool instead of the tweezers. Finally, the last avatar was a hand holding a pair of tweezers. Subjects explored the Virtual Reality (VR) environment from the avatar first-person perspective employing an HTC VIVE PRO head-mounted display (HMD) (www.vive.com). The HMD has a 110° field of view, a resolution of 1440 x 1600 per eye and internal sensors to track head movements. To control the virtual hands in real time the Leap Motion infrared camera was used (www.ultraleap.com) in combination with inverse kinematics techniques to animate the arms. The vibrotactile feedback for the CCT was delivered using a custom haptic device.

The haptic device was realized with: (i) an Adafruit Stereo 20W Class D Audio Amplifier MAX9744; (ii) two voice coils Haptuator Planar of TactileLabs; (iii) a LiPo battery at 3.7V and 400mAh to supply power to the interface; (iv) an Adafruit PowerBoost 500 Charger to step-up to 5V the LiPo provided voltage and to recharge the battery; (v) an ABS 3D printed case to assemble all components in a compact space.

The audio amplifier drives the two Haptuator Planar through amplification of the two-channel input audio signal. The MAX9744 is cool-running, no heat sinks are required, and its high efficiency, up to 93%, makes it great for portable or battery-powered devices.

Haptuator Planar is a miniaturized planar voice coil. In addition to a high bandwidth of 50-500Hz, the Haptuator Planar consists of a soft surface specifically designed to directly stimulate the skin. The 6 mm low profile and the 1.8 g light weight allow it to be embedded in small devices. Despite the small size, the Haptuator Planar can provide an output acceleration of 4G at 125Hz when powered at 1V and with a 5g extra load (20g total weight).

No audio feedback was delivered.

#### Task and experimental procedure

During Experiment 1, participants performed two types of tasks: a Cross-modal Congruency task (CCT), and a Motor Task (MT). Before the experiment, participants were asked to become familiar with the environment and the virtual scene was adjusted to match the height of the participant. During the MT, they were asked to pop virtual colored spheres using either the virtual hand or the tool to become confident with the motor task. The goal of their task was to pop the indicated color bubble (i.e., sphere), using the pinch of their virtual fingers or virtual tweezers, from a set of 12 different colored spheres. The target color was suggested by a sphere that appeared at the beginning of the trial within a transparent cube placed on the virtual desk. During the CCT (adapted from^44^), a small yellow dot flashing near participants’ virtual index or thumb (or the upper/lower branch of the tool) appeared. Simultaneously, participants felt a vibration either on their real index or thumb. Participants were instructed to respond as to where the vibration was felt. They responded by pressing the left mouse button or the mouse right button with their left hand. During congruent trials, the light appeared where the participant felt the vibration, while in incongruent trials the visual and the vibrotactile stimuli appeared on different fingers. Errors were counted as follows: 1) the participant indicated the wrong location of vibration (e.g., the vibration was on the right index, but the participant pressed the right mouse button with his/her thumb); 2) there was no response before 1800 ms or before 200 ms (this kind of answer was too rapid, indicating an anticipation based on probability instead of the right processing of the stimulus). Only the first press of the mouse was counted as a response. During the CCT participants were instructed to look at the virtual effector and stay still.

Regarding the MT, as soon as the cross-modal task was concluded, the participant was instructed to press a gray virtual sphere with his/her left virtual hand to start the block. First, a colored sphere appeared inside a virtual transparent green box, indicating the color of the target sphere to pop, at the same time a total of 12 spheres appeared above this box (4 per color). The participant was instructed to reach a sphere of the same color as the target one and pop it with a pinching motion. The spheres disappeared after 4000 ms.

The crossmodal congruency task consisted of 9 blocks in total with 10 trials per block. The MT task consisted of 8 blocks with 12 trials per block.

The hand and tool conditions were balanced between subjects (Figure 1A).

In Experiment 2, participants underwent 9 blocks of CCT performed with the virtual bionic tool (i.e., a pair of tweezers) (Figure 1B). In Experiment 3 participants were asked to perform CCT (9 blocks) with 3 different virtual bionic effectors (i.e., tweezers, a wrench, a hand) (Figure 1C). In Experiment 4, participants performed 9 blocks of CCT and 8 blocks of MT with two different effectors (i.e., a bionic tool and a hand holding a pair of tweezers) (Figure 1D). In addition, we administered participants a questionnaire to measure embodiment adapted from.^39^ The items were adapted for the purposes of the present study and translated in Italian. We dropped two items because they did not comply with the present experimental design, thus in total the questionnaire was made up of 16 items. The questionnaire assesses embodiment on three different scales: Embodiment (i.e., embodiment of the virtual effector), Disembodiment (i.e., the feeling of control and ownership toward participants’ real hand) and Physical sensations (i.e., pleasant/unpleasant sensations felt on participants’ real hand). Participants had to state their agreement or disagreement for each item on a Likert scale ranging from −3 (Strongly disagree) to 3 (Strongly agree).

### Quantification and statistical analysis

All analyses were performed with R (R Core Team, 2021). We used linear mixed models to evaluate the fixed and the random effect predictors on the reaction times (RTs), for both the CCT and MT. For the score on the MT and errors in CCT we used a generalized linear mixed model (Brown and Prescott, 2015; Bolker 2015). In experiment 1 we run a linear mixed model on RTs of the CCT with Effector (i.e., virtual hand and virtual prosthesis) and Type of trial (congruent and incongruent) as fixed effects. The model also included the interactions between them. In all the experiments, we used dummy coding to account for the effects of Effector and Type of trial. This means that each of the two parameters estimates the difference in RTs with respect to the baseline (hand-congruent in experiment 1). The interaction term between Effector and Type of trial accounts for the difference in CCE between the two effectors. We included participants as a random effect in the model. To model the errors in the task we used a generalized linear mixed model (with a log link function) with Effector and type of trial as fixed effects, and participants as random effect. The estimate of the parameter corresponds to the effect size in the model.

For the MT we analyzed the performance in terms of score using a generalized linear mixed model with Effector as fixed effect and participants as random effect parameter. For the RTs we used a linear mixed model with Effector as the fixed effect and participants as the random effect.

In experiment 2, for RTs we used the linear mixed model with Type of trial (i.e., congruent and incongruent) as the fixed effect and participant as the random effect. To analyze the errors we used the same model of Experiment 1 with only the fixed effect of Type of trial and participants as random effect.

In experiment 3 we analyzed RTs with a linear mixed model using the Effector (i.e., hand, tweezers, wrench) and Type of trial (i.e., congruent and incongruent) as fixed effects. The same factors were used in a generalized linear mixed model for the analysis of errors.

Analyses of experiment 4 were performed using the same models and fixed effects of experiment 1.

All results (estimate, standard error, p-value) are reported in Table 1.

In all analyses the significance level was set at p < 0.05 (∗p < 0.05, ∗∗p < 0.01, ∗∗∗p < 0.001).

Regarding the questionnaire (experiment 4), we performed separate Wilcoxon tests for the three dimensions (i.e., Embodiment, Disembodiment, Physical sensations) to assess differences on the scale scores between the two end-effectors.

## Footnotes

Supplemental information can be found online at https://doi.org/10.1016/j.isci.2024.109937.
