# Supplementary material for: Rewiring the evolution of the human hand: How the embodiment of a virtual bionic tool improves behavior
Source: iScience. 2024 Jun 6;27(6):109937. doi: 10.1016/j.isci.2024.109937 (PMC11270032; doi:10.1016/j.isci.2024.109937)
Supplement: Document S1. Figures S1, S2, and Methods S1 [file mmc1.pdf]

iScience, Volume *27*

Supplemental information

Rewiring the evolution of the human hand: How the embodiment of a virtual bionic tool improves behavior

Matteo Marucci, Ottavia Maddaluno, Colleen Patricia Ryan, Cristina Perciballi, Simona Vasta, Simone Ciotti, Alessandro Moscatelli, and Viviana Betti

Supplementary Information

Figure S1. Mean Reaction Times and Errors for congruent and incongruent trials in the CCT: experiment 1 and 2. Related to Figure 2 and Figure 3. Upper panel: results of experiment 1. Here depicted mean RTs for congruent and incongruent trials for the virtual hand and the bionic tool (on the left). Participants were slower during incongruent trials than for congruent ones. Crucially, the difference in reaction times between the two types of trials was bigger when participants used the virtual bionic tool to perform the task. On the right, mean number of errors. Participants committed more errors during incongruent trials when performing the CCT with the bionic tool. Lower panel: RTs and errors for experiment2. During incongruent trials RTs were slower and the number of errors was bigger. (*p < 0.05, **p < 0.01, ***p < 0.001).

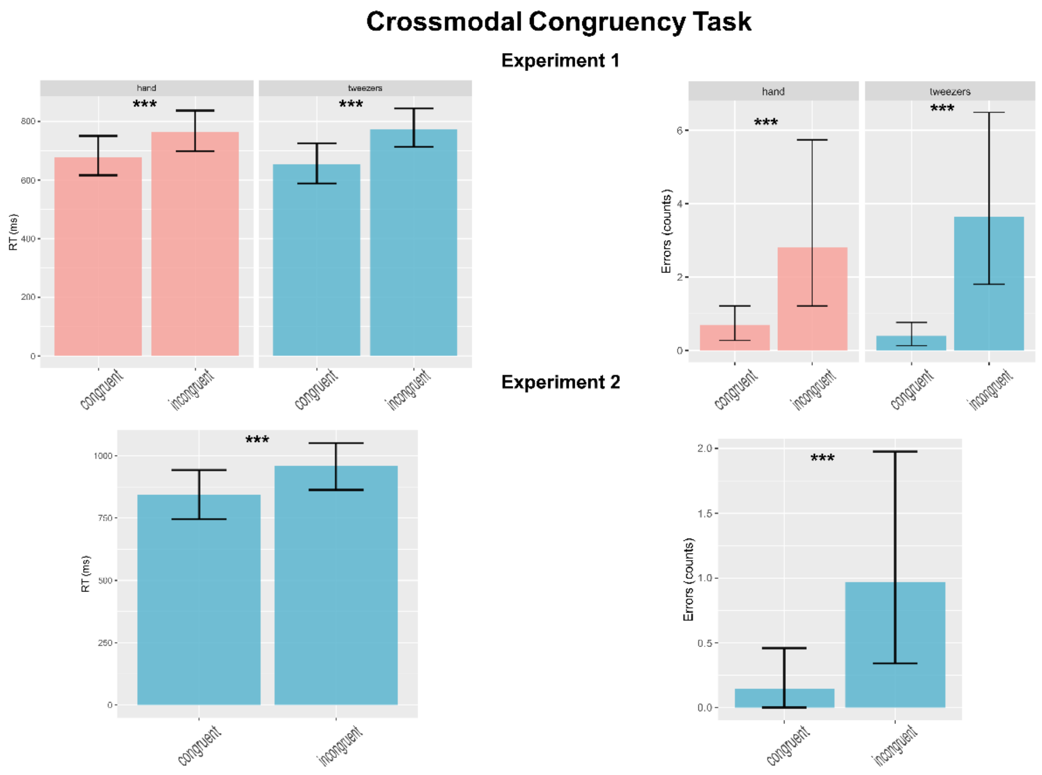

Figure S2. Mean Reaction Times and Errors for congruent and incongruent trials in the CCT: experiment 3 and 4. Related to Figure 3 and Figure 4. Upper panel: mean RTs and number of errors for the different end-effectors: *i.e.*, virtual hand and virtual bionic tool (either a pair of tweezers or a wrench). On average participants were slower and committed more errors on incongruent trials, regardless of the effector. Lower panel RTs and errors for experiment 4. RTs are higher in incongruent trials than congruent ones. Likewise, the number of errors during the incongruent condition was higher than in the congruent one. (*p < 0.05, **p < 0.01, ***p < 0.001).

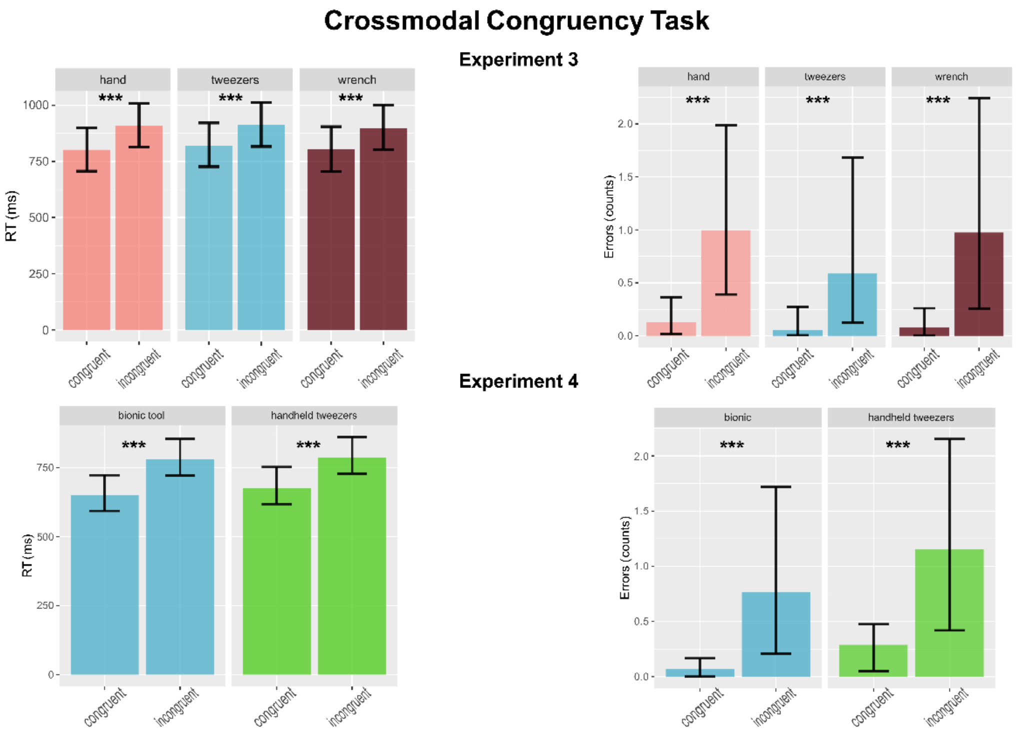

Methods S1. Embodiment questionnaire (adapted from Romano et al. 2021) used to assess embodiment in experiment 4. Related to Figure 5.

Embodiment Scale (ES)

“Durante il Blocco...

1(E1)	…sembrava guardassi la mia mano invece che uno strumento virtuale	-3 -2 -1 0 +1 +2 +3 _______
2(E2)	…sembrava che lo strumento virtuale cominciasse ad assomigliare alla mia vera mano	-3 -2 -1 0 +1 +2 +3 _______
3(E3)	…sembrava che lo strumento virtuale appartenesse a me	-3 -2 -1 0 +1 +2 +3 _______
4(E4)	...sembrava che lo strumento virtuale fosse la mia mano	-3 -2 -1 0 +1 +2 +3 _______
5(E5)	...sembrava che lo strumento virtuale fosse parte del mio corpo	-3 -2 -1 0 +1 +2 +3 _______
6(E6)	...sembrava che la mia mano fosse nel punto in cui si trovava lo strumento virtuale	-3 -2 -1 0 +1 +2 +3 _______
7(E7)	...sembrava che lo strumento virtuale fosse nel punto in cui si trovava la mia mano	-3 -2 -1 0 +1 +2 +3 _______
9(E9)	...sembrava che avrei potuto muovere lo strumento virtuale se avessi voluto	-3 -2 -1 0 +1 +2 +3 _______
10(E10)	...sembrava che avessi il controllo dello strumento virtuale	-3 -2 -1 0 +1 +2 +3 _______
11(D1)	...sembrava che non fossi in grado di muovere la mia mano	-3 -2 -1 0 +1 +2 +3 _______
12(D2)	...sembrava che non potessi davvero dire dove fosse la mia mano	-3 -2 -1 0 +1 +2 +3 _______
13(D3)	...sembrava che la mia mano fosse scomparsa	-3 -2 -1 0 +1 +2 +3 _______
14(D4)	...sembrava che la mia mano fosse fuori dal mio controllo	-3 -2 -1 0 +1 +2 +3 _______
15(D5)	...sembrava che la mia mano si stesse muovendo verso lo strumento virtuale	-3 -2 -1 0 +1 +2 +3 _______
16(D6)	...sembrava che lo strumento virtuale si muovesse verso la mia mano	-3 -2 -1 0 +1 +2 +3 _______
18(P2)	...avevo la sensazione di spilli e aghi nella mia mano	-3 -2 -1 0 +1 +2 +3 _______

“Durante il Blocco...

1(E1) …sembrava guardassi la mia mano invece che una mano virtuale che teneva le pinzette

2(E2) …sembrava che la mano virtuale che teneva le pinzette cominciasse ad assomigliare alla mia vera mano

3(E3) …sembrava che la mano virtuale che teneva le pinzette appartenesse a me

4(E4) ...sembrava che la mano virtuale che teneva le pinzette fosse la mia mano

5(E5) ...sembrava che la mano virtuale che teneva le pinzette fosse parte del mio corpo

6(E6) ...sembrava che la mia mano fosse nel punto in cui si trovava la mano virtuale che teneva le pinzette

7(E7) ...sembrava che la mano virtuale che teneva le pinzette fosse nel punto in cui si trovava la mia mano

9(E9) ...sembrava che avrei potuto muovere la mano virtuale che teneva le pinzette se avessi voluto

10(E10) ...sembrava che avessi il controllo della mano virtuale che teneva le pinzette

11(D1) ...sembrava che non fossi in grado di muovere la mia mano

12(D2) ...sembrava che non potessi davvero dire dove fosse la mia mano

13(D3) ...sembrava che la mia mano fosse scomparsa

14(D4) ...sembrava che la mia mano fosse fuori dal mio controllo

15(D5) ...sembrava che la mia mano si stesse muovendo verso la mano virtuale che teneva le pinzette

16(D6) ...sembrava che la mano virtuale che teneva le pinzette si muovesse verso la mia mano

18(P2) ...avevo la sensazione di spilli e aghi nella mia mano
